# Extracapsular dissection for clinically benign parotid lumps: reduced morbidity without oncological compromise

**DOI:** 10.1038/sj.bjc.6601281

**Published:** 2003-10-28

**Authors:** M McGurk, B L Thomas, A G Renehan

**Affiliations:** 1 Salivary Gland Service, Department of Oral and Maxillofacial Surgery, Floor 23 Guy's Tower, Guy's Hospital, London Bridge, London SE1 9RT, UK; 2 Department of Surgery, Christie Hospital NHS Trust, Manchester M20 4BX, UK

**Keywords:** parotid gland, extracapsular dissection, carcinoma, facial nerve palsy, survival

## Abstract

Previous studies have shown that extracapsular dissection (ECD) is an alternative approach to superficial parotidectomy (SP) for pleomorphic adenoma parotid tumours, associated with low recurrence rates equal to those following SP, but with significantly reduced morbidity. However, if a malignant tumour masquerades as a clinically benign lump, this approach may be inappropriate. This study addressed this question by analysing the outcome of 821 consecutive patients with parotid tumours treated at one centre over 40 years and with a median 12 (range 5–30) years follow-up. Tumours were classified as ‘simple’ (discrete, mobile, < 4 cm: *n*=662) and ‘complex’ (deep, fixed, facial nerve palsy, ⩾4 cm: *n*=159). Among the ‘simple’ or clinically benign tumours, 503 patients underwent ECD; 159 patients underwent SP. In all, 32 (5%) clinically benign cases were subsequently revealed as malignant histologies (ECD, 12; SP, 20). For each group, 5- and 10-year cancer-specific survival rates were 100 and 98%, respectively. There were no differences in recurrence rates when subanalysed by surgical groups, but ECD was associated with significantly reduced morbidity (*P* < 0.001). This study demonstrates that ECD is a viable alternative to superficial parotidectomy for the majority of parotid tumours, associated with reduced morbidity without oncological compromise.

Parotid tumours are uncommon, with the majority presenting as discrete lumps arising within the superficial portion of the gland (Renehan *et al*, 1996). Conventional teaching prescribes removal of these tumours by superficial parotidectomy (SP), which encompasses facial nerve identification and *en bloc* removal of the superficial portion of the gland (Langdon, 2001). Extracapsular dissection (ECD) is an alternative approach to the removal of such lumps involving meticulous dissection immediately outside the tumour capsule while still preserving the facial nerve (Anderson, 1975; Gleave, 1995), and is distinct from enucleation. Based on the traditional view that many parotid tumours (notably pleomorphic adenomas) breach their capsule and so are theoretically at risk of recurrence from surgery close to the capsule (the ‘tumour bud’ concept) (Thackray and Lucas, 1974; Lawson, 1989, Naeim *et al*, 1976), ECD has been received with caution. However, we have previously demonstrated in 475 patients with pleomorphic adenomas that ECD is associated with long-term low recurrence rates (< 2%) comparable with SP, but fewer complications (McGurk *et al*, 1996). Thus, in the practice of treating parotid pleomorphic adenomas, ECD reduces morbidity without oncological compromise. However, this procedure may not be appropriate for malignant tumours. The potential risk in ECD is encountering a malignant tumour masquerading as a benign lump – if this occurrence is common and the subsequent course of the cancer is adversely affected, it would prohibit the use of ECD as an alternative to SP for a simple parotid lump. This study addressed this question by analysing the outcome of 821 consecutive patients with parotid tumours treated at one centre over 40 years, and in particular, focuses on outcome when the histology was a carcinoma.

## METHODS

### Patients and pathology

Between 1952 and 1992, 821 patients with previously untreated epithelial parotid neoplasia were treated at the Christie Hospital, Manchester (Renehan *et al*, 1996, 1999; Gleave *et al*, 1979). The preoperative diagnosis was made on clinical grounds; fine needle aspiration cytology was not used. Tumours were classified by clinical criteria into (i) ‘simple’, which are discrete, mobile, and less than 4 cm in diameter (for the purpose of this paper, this is synonymous with ‘clinically benign’); and (ii) ‘complex’, which are greater than 4 cm and/or demonstrate fixity, facial nerve involvement, palpable cervical nodes, or deep lobe involvement (McGurk *et al*, 1995, 2001). For tumours with a malignant histology, simple tumours equated to AJCC Stage I disease. Complex tumours were not treated by ECD, and excluded from the analysis. The histological diagnoses were updated in the mid-1970s in line with the WHO classifications (Thackray and Sobin, 1972; Seifert and Sobin, 1991). Median follow-up was 12 (range 5–32) years.

### Treatment

Among the ‘simple’ tumours, 503 patients underwent ECD, 159 patients underwent SP. The choice of technique was made on clinical criteria, namely mobility at surgery after raising the skin flap. This procedure has been described in detail elsewhere (Gleave, 1995). In this technique, the plane of dissection is within a compartment of loose areolar tissue approximately 2–3 mm from the tumour. This contrasts sharply with enucleation, which breaches the capsule and removes the tumour within. Postoperative adjuvant radiotherapy (RT) (McGurk *et al*, 1996; Renehan *et al*, 1999) was used selectively, either for spillage of benign tumour or for those malignant tumours with a positive margin, spillage, adenoid cystic or high-grade carcinomas.

### Statistical analysis

Comparisons of means were by Student's *t*-test; proportions were compared by χ^2^-tests or Fisher's exact test, where expected frequencies were less than five. Cancer-specific survival and recurrence rates were calculated by the Kaplan–Meier method and differences tested by log-rank (STATA version 7.0, College Station, CA, USA).

## RESULTS

### Basic characteristics

The baseline characteristics for the 821 tumours are outlined in Table 1

**Table 1:** Characteristics of parotid tumours, Christie Hospital (1952–1992)

	**Simple**	**Complex**
Total number	662	159
Median age (range) (years)	47 (5–87)	58 (25–85)
Males	267 (40)	83 (50)
Females	395	82

*Benign histology*	630 (95)	50 (31)
Pleomorphic adenoma	487	45
Warthin's tumour	131	5
Monomorphic adenomas	12	0

*Malignant histology*^a^	32 (5)	109 (69)
Acinic cell carcinoma	7	6
LG mucoepidemoid carcinoma	13	11
HG mucoepidermoid	—	3
Basal cell adenocarcinoma	—	1
Papillary cystoadenocarcinoma	2	2
Mucinous adenocarcinoma	—	1
Adenocarcinoma, NOS	—	16
Adenoid cystic carcinoma	5	28
Epithelial–myopeithelial ca.	—	1
Ca. ex-pleomorphic adenoma	4	13
Squamous cell carcinoma	—	7
Undifferentiated carcinoma	1	20

a For malignant tumours, ‘simple’ lumps are equivalent to AJCC Stage I disease.

Values in parentheses are percentages unless otherwise indicated. See text for definitions of ‘simple’ and ‘complex’. NOS=not otherwise specified; ca.=carcinoma. LG=low-grade; HG=high-grade.

. The majority (662 or 81%) presented as ‘simple’ lumps, of which only 32 or 5% proved to carcinomas. By contrast, two-thirds of ‘complex’ tumours had a malignant histology. The pattern of malignant histological types differed between ‘simple’ and ‘complex’ tumours: notably, two-thirds of ‘simple’ lumps with subsequent malignant histology were either acinic cell or low-grade mucoepidermoid carcinoma.

### Survival and recurrence rates

In the 32 patients with ‘simple’ lumps, which proved to be carcinomas, 12 underwent ECD and 20 underwent SP. Following surgery, over half of these patients received radiotherapy (ECD, 7/12: SP, 12/20)–the indications are shown in Table 2

**Table 2:** Indications for postoperative radiotherapy among 19 patients with stage I cancers

	**Extracapsular dissection (*n*=7)**	**Superficial parotidectomy (*n*=12)**
Positive tumour margin	2	0
Close tumour margin	3	7
Tumour spillage	0	2
High-grade cancer	2	3
Adenoid cystic carcinoma	1	3

For some tumours, there may have been more than one indication for postoperative radiotherapy.

. While these surgical groups were not randomly determined, the clinicopathological characteristics were broadly comparable (Table 3

**Table 3:** Clinicopathological characteristics of 32 stage I carcinomas

	**Extracapsular dissection (*n*=12)**	**Superficial parotidectomy (*n*=20)**	***P*-value^a^**
Age (mean) (s.d.)	44 (21)	52 (22)	0.33
Male : female	5 : 7	10 : 10	
Size less than 2 cm	4 (33)	5 (25)	0.70

*Histology*			
Mucoepidermoid ca.	7 (58)	6 (30)	
Acinic cell ca.	1	6 (30)	
Adenoid cystic ca.	1	4 (20)	
Other carcinomas	3 (25)	4 (20)	

*Malignant grade*			
Grade I	9 (75)	13 (65)	0.70
Grade II	1	3 (15)	
Grade III	2	3 (15)	
Positive tumour margins	2	0	1.00
Close tumour margins	3 (25)	8 (40)	0.46
Perineural invasion	0	1	
Tumour spillage	0	2	1.00

a Student's *t*-test, χ^2^, and Fisher's exact tests as appropriate.

**Figure 1:**
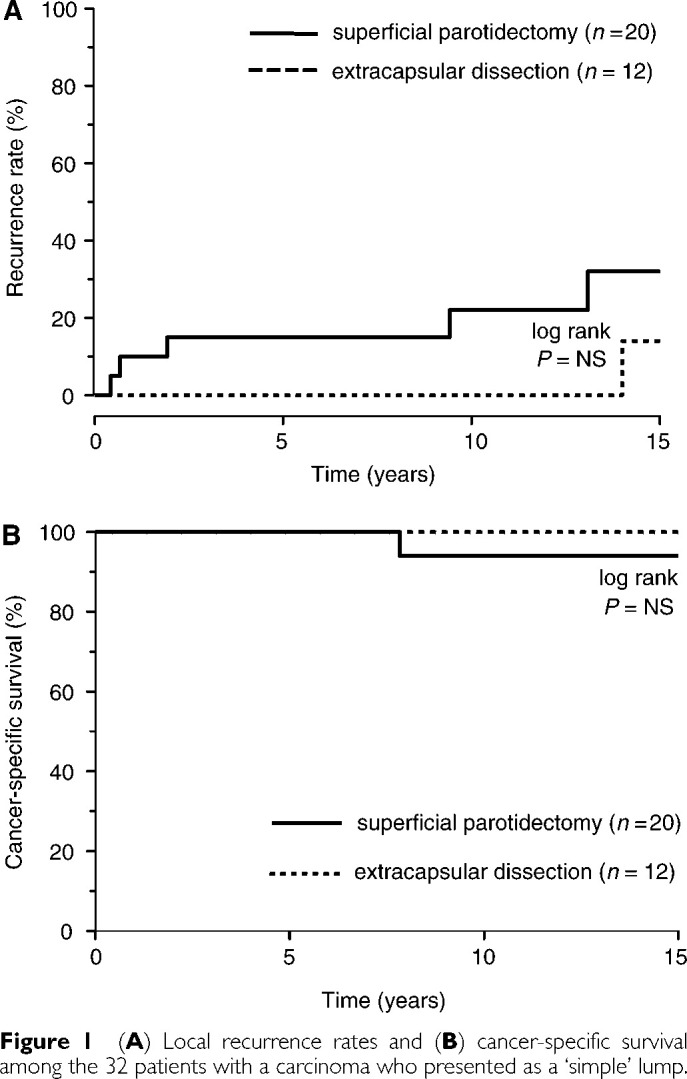
(**A**) Local recurrence rates and (**B**) cancer-specific survival among the 32 patients with a carcinoma who presented as a ‘simple’ lump.

Values in parentheses are percentages. s.d.=standard deviation.

). The 5- and 10-year cancer-specific survival rates were 100 and 98%, respectively for ECD and SP (Figure 1A

). Local recurrences developed in six cases (ECD, 1: SP, 5), with no statistical difference by log-rank testing between the two groups (Figure 1B). Only one of the six cancer recurrences occurred in a patient who had received both surgery and radiotherapy (Table 4

**Table 4:** Details of six recurrences from 32 cases with AJCC stage I malignant disease

**Summary of treatment, pathology, and outcome**	
Case 1	73 year, male; SP + postoperative RT; ca ex-PSA, Grade III; close margin; LC at 23 months; died unrelated cause
Case 2	10 year, female; SP, no RT; mucoepidermoid ca.; Grade I; clear margins; LA at 5 months; salvaged; disease-free at 12 years
Case 3	77 year, female; SP, no RT; adenoid cystic ca.; Grade II; close margin; LC at 8 months; died of disease
Case 4	59 year, female; SP, no RT; adenoid cystic ca.; Grade II; clear margin; LC at 157 months; salvaged
Case 5	59 year, male; SP, no RT; papillary adenocarcinoma; Grade I; clear margin; LC at 113 months; salvaged; disease free at 5 years
Case 6	63 year, female; EDC, no RT; papillary adenocarcinoma; Grade I; clear margin; LC at 168 months; salvaged; disease-free at 7 years

SP=superficial parotidectomy; EDC=extracapsular dissection; RT=radiotherapy; ca. ex-PSA=carcinoma ex-pleomorphic adenoma; LC=local recurrence.

); thus, radiotherapy *per se* may have been a confounding factor in determining recidivism.

Of the 630 patients with ‘simple’ lumps and benign histologies, there were 10 recurrences at 15 years. Eight recurrences occurred after 491 ECDs (1.7% at 15 years by life-table analysis); two recurrences occurred after 139 SPs (1.8% at 15 years by life-table analysis).

### Morbidity

The complications by surgical groups and histological categories are shown in Table 5

**Table 5:** Complications after treatment of benign and malignant histologies

	**Benign histologies**			**Malignant histologies**		
	**ECD (*n*=491)**	**SP (*n*=139)**	***P*-value^a^**	**ECD (*n*=12)**	**SP (*n*=20)**	***P*-value^a^**
*Facial nerve palsy*						
Permanent	8 (1.6)	2 (1.4)	NS	1 (8)	3 (15)	NS
Transient	48 (10)	45 (32)	< 0.001	2 (16)	3 (15)	NS
Frey's syndrome	25 (5)	45 (32)	< 0.001	2 (16)	3 (15)	NS
Amputation neuroma	17 (3)	22 (16)	< 0.001	2 (16)	4 (20)	NS
Salivary fistula	3 (0.6)	0	NS	0	0	NS

a χ^2^ and Fisher's exact tests as appropriate.

Values in parentheses are percentages. NS=not significant.

. Among the 630 patients with a ‘simple’ tumour and benign pathology, ECD was associated with significantly reduced morbidity compared with SP, including transient facial palsy (*P* < 0.001), Frey's syndrome (*P* < 0.001), and amputation neuroma (*P* < 0.001) (Table 5). Salivary fistulae developed without obvious causes in three patients treated by ECD but resolved spontaneously within 3 months. For the patients with ‘simple’ tumours and malignant histologies, complication rates were generally higher, but this was based on small patient numbers. There were no apparent differences between ECD and SP.

## DISCUSSION

This study has demonstrated that the majority of parotid tumours present as a clinically benign lump, and of these, only 5% subsequently prove to be carcinomas. This study establishes ECD as a viable alternative surgical approach to superficial parotidectomy in such tumours, for it has the advantage of reduced morbidity without untoward effects on oncological outcome.

The advantages of this study were large numbers of patients treated with a similar surgical philosophy, and followed for a long period. Thus, we were able to show that out of 662 clinically benign parotid lumps, SP was avoided in 503 patients, and replaced with a more conservative procedure carrying significantly less morbidity. In adopting this conservative approach, there was a diagnostic error rate of 32/662, with 12 patients treated ‘inappropriately’ by ECD. The question was whether these ‘errors’ resulted in a poor outcome, but this was not the case. The authors accept that the 12 patients should ideally have been treated by some form of parotidectomy, and in such circumstances, seven patients may have avoided RT–these represent the penalty paid for saving 503 patients the need to undergo parotidectomy.

The merit of classifying discrete parotid lumps into ‘simple’ or ‘complex’ is confirmed in this study. Using clinical judgement alone, the assessment of 662 simple lumps (‘benign tumours’) was correct in 95% of the cases. Following a final step of clinical assessment at surgery, 12 of the 32 malignant tumours were removed by ECD rather than SP (a clinical diagnostic error rate of 1.8%). This equates to a test sensitivity of 93% and a positive predictive value of 95% (Altman and Bland, 1994). In theory, if clinical assessment is supplemented with fine needle aspiration cytology (McGurk and Hussain, 1997), these predictive values may increase.

Only 5% of clinically benign parotid tumours were carcinomas and notably two-thirds of these were low-grade cancers (acinic cell and low-grade mucoepidermoid carcinomas). Half required postoperative radiotherapy but this did not represent overtreatment of Stage I disease (Frankenthaler *et al*, 1991). Irrespective of the surgical approach, the long-term prognosis in this group was good. A similar experience was reported by Nnochiri *et al* (1990), who encountered 20 (4%) carcinomas unexpectedly in a series of 539 otherwise unremarkable parotid tumours. These tumours were treated surgically as nonmalignant lumps, yet subsequently followed a benign course.

As long-term low recurrence rates are now the norm for parotid pleomorphic adenomas, there is an emerging trend towards low morbidity surgery. Series employing either total parotidectomy or formal superficial parotidectomy report high rates of transient facial nerve palsy (15–70%) and Frey's syndrome (13–66%) (reviewed in Langdon, 2001). Recent studies have advocated a more conservative parotidectomy, partial superficial parotidectomy, and report lower transient facial nerve rates (20–33%) and Frey's syndrome rates (7–20%) (Yamashita *et al*, 1993; Helmus, 1997; Leverstein *et al*, 1997; Snow, 2001). Still lower rates of morbidity have been reported, following ECD (Anderson, 1975; Martis, 1983; Prichard *et al*, 1992) with 3–11% transient facial nerve palsy and 0–5% Frey's syndrome. The very low rates of gustatory sweating after ECD are presumably due to less disruption of the parotid tissue. ECD also avoids the unsightly complication of retromandibular depression frequently observed after superficial parotidectomy.

As the needs for reducing morbidity and maintaining facial aesthetics increase, ECD represents the current limit of conservative parotid surgery. A common feature of all minimally invasive therapies is that the technique leaves little room for error. With acceptance of these limitations, the findings of this study demonstrate that ECD is a scientifically valid and oncologically safe approach to the management of the clinically benign parotid lump.
